# Changes and Diurnal Variation of Visual Quality after Orthokeratology in Myopic Children

**DOI:** 10.1155/2018/3174826

**Published:** 2018-10-15

**Authors:** Hao-Chen Guo, Wan-Qing Jin, An-Peng Pan, Qin-Mei Wang, Jia Qu, A-Yong Yu

**Affiliations:** The Eye Hospital of Wenzhou Medical University, Wenzhou, Zhejiang, China

## Abstract

**Purpose:**

To assess the changes and the diurnal variation of visual quality after orthokeratology in myopic children.

**Methods:**

Forty-four eyes of 22 subjects with a mean age of 10.55 ± 1.53 years (8 to 14 years) were enrolled in this prospective study. Their spherical equivalent ranged from −1.25 to −4.25 diopters (D) and astigmatism was less than 1.00 D. Parameters including corneal curvature, ocular objective scatter index (OSI), the modulation transfer function (MTF), root mean square of ocular and corneal wavefront aberrations, and contrast sensitivity function (CSF) were measured before and at two time points during the same day after 1 month of orthokeratology.

**Results:**

After orthokeratology, uncorrected visual acuity (UCVA) and spherical equivalent were significantly improved from baseline (*P* < 0.001), and their diurnal variation was not significant (*P*=0.083, 0.568). OSI increased from 0.29 ± 0.15 to 0.65 ± 0.31 (*P* < 0.001). MTF decreased significantly (*P* < 0.01). Corneal curvature and ocular total aberration decreased (*P* < 0.001), while the ocular and corneal higher-order aberration increased significantly (*P* < 0.01). The CSF under photopic condition decreased at 3 cpd (*P*=0.006) and increased at 18 cpd (*P*=0.012). The diurnal variation of CSF at 18 cpd under mesopic and high glare conditions and at 12 cpd under photopic condition was significant (*P*=0.002, 0.01, 0.017).

**Conclusions:**

Orthokeratology can effectively improve UCVA and high spatial frequency CSF by decreasing the low-order aberrations. However, MTF and CSF at low spatial frequency decreased because of the increase of intraocular scattering and high-order aberrations. Meanwhile, CSF at high spatial frequency fluctuates significantly at two times during the same day after 1 month orthokeratology.

## 1. Introduction

Orthokeratology involves wearing of specially designed gas-permeable contact lenses which temporarily reshape corneal contour [1]. This procedure can offer patients useful vision during waking hours without involving additional corrective devices, such as spectacles or daily wear contact lenses. However, unstable vision during waking hours, transient light distortion under low-light condition, and dissatisfied night vision were reported by certain patients [2]. Several studies have demonstrated that overnight orthokeratology may increase corneal and ocular higher order aberrations [3–5] and decrease contrast sensitivity function (CSF) [4–6]. Furthermore, several short-term studies have reported that the influence of orthokeratology on refraction and visual acuity gradually diminished during the day once the lens was removed [7–10], which may cause uncomfortable visual experience as mentioned above. These studies focus mostly on wavefront aberration, visual acuity, and refraction. However, these assessments are insufficient to fully understand the effects of orthokeratology on visual quality because retinal image is affected not only by ocular aberration but also by intraocular scattering [11, 12].

Research based on double-pass technique have revealed that the retinal image quality may be overestimated by aberrometric techniques which often failed to take the effect of diffuse light (dispersion or scattering) into account, and the double-pass system has been proven to be a useful tool for comprehensive evaluation of optical quality of the eye because it can provide parameters that included intraocular scattering [12–14]. There were few studies using double-pass technique to evaluate the visual quality after orthokeratology. Jeon et al. [15] used the double-pass system in 13 patients (24 eyes) and found that the intraocular scattering increased after 1 month of orthokeratology lenses wear. However, that study did not involve the diurnal variation of visual quality. Recent studies suggested that the combined effect of ocular aberration and intraocular scattering on the visual quality was not a simple summation, and the peripheral aberration could compromise partial effect of scattering [16]. The study on contact lenses using the double-pass technique found that corneal swelling caused increased intraocular scattering, resulting in a significant impact on the optical quality of the eye [17]. Currently, limited data were available on the changes and diurnal variation of comprehensive visual quality after orthokeratology in myopic children. This study aimed to provide information on changes and diurnal variation of visual quality after orthokeratology by analyzing the data of refraction, intraocular scattering, corneal topography, wavefront aberration, CSF, and subjective questionnaire. The comprehensive measurements of these changes are essential for a better understanding of the impact of orthokeratology on vision, especially in children.

## 2. Methods

In this prospective study, 44 eyes of 22 myopic patients (9 boys, 13 girls) with a mean age of 10.55 ± 1.53 yrs (mean ± standard deviation, range: 8 to 14 yrs) were enrolled. Spherical equivalent ranged from −1.25 to −4.25 D (−2.81 ± 0.87 D), and astigmatism was less than 1.00 D (0.48 ± 0.21 D). The best corrected visual acuity (BCVA) was 20/20 or better. After 1 month of orthokeratology, only eyes with an uncorrected visual acuity (UCVA) of 20/20 or better were included. Subjects with a history of contact lens wear or any current ocular or systemic disease such as a significant dry eye, papillary conjunctivitis, keratoconus, corneal dystrophies, and corneal opacities that could affect ocular physiology were excluded. This study was in accordance with the tenets of the Declaration of Helsinki and approved by the Ethics Committee at the Eye Hospital of Wenzhou Medical University. Informed consent was obtained from each subject and patient.

The baseline measurements were taken in both eyes before orthokeratology lens fitting in the morning at the initial visit, including visual acuity (logarithmic visual acuity chart, GB 11533—1989), manifest refraction (Phoroptor, Phorovist 200), corneal curvature (Keratometer, OM-4), noncontact tonometer (Nidek NT-2000), corneal topography (E300 Corneal Topographer), corneal endothelial count (Topcon, SP-2000P), and axial length (IOL-MASTER, Zeiss). For each subject, the best suitable orthokeratology lens was chosen from three brands (Table 1) according to the different fitting situations. The subjects were recommended to wear the lenses 7 nights a week and at least 8 hours per night to ensure the best situation of UCVA. All the subjects were monitored by the same experienced doctor. One month after orthokeratology, the measurements were taken immediately following lens removal in the morning and 8 hours later in the afternoon during the same day. At every visit, all the measurements were taken within 30 minutes to minimize the fluctuation of each parameter.

### 2.1. Double-Pass Measurements

The objective parameters of optical quality were measured by a double-pass optical quality analysis system (OQAS II, Visiometrics S.L., Tarrasa, Spain). A single light source produced by a 780 nm laser beam adequately filtered and collimated was used as a starting point. The beam image was projected onto the eye retina. When it reflects on the retina, the light crosses the ocular medium twice and OQAS II analyses the size and shape of the reflected point of light [14]. Room illumination was kept low during the measurement to ensure a natural pupil diameter larger than 5 mm without dilation. The measurements were taken with artificial pupil diameters of 3, 4, and 5 mm, respectively. Astigmatism >0.50 D was corrected by using external ophthalmic cylindrical lenses. The main parameters provided by the double-pass system were the modulation transfer function (MTF) cut-off frequency in cycles per degree (cpd), Strehl ratio (SR), OQAS values (OVs) 100%, 20%, and 9%, objective scatter index (OSI), and tear film mean OSI (TFM-OSI). Three consecutive measurements were obtained for each eye and the mean value was calculated.

### 2.2. Wavefront Aberration Measurements

**Figure 1:**
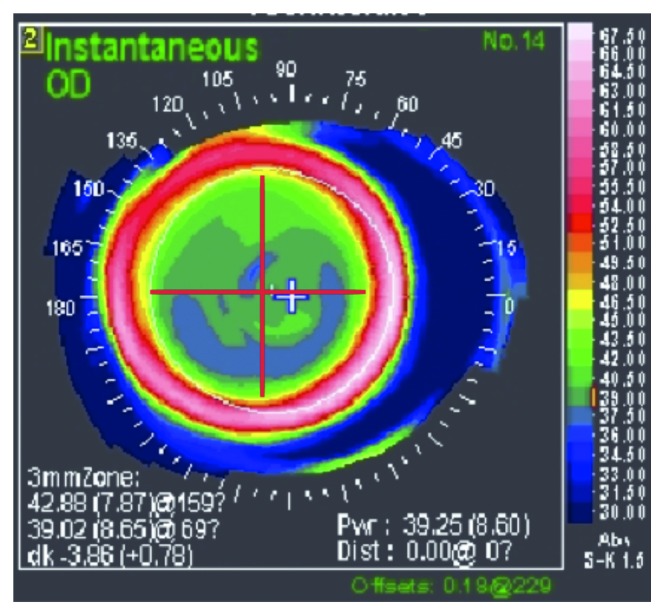
Instantaneous map. ^∗^The instantaneous map calculates the corneal curvature radiuses from the shape between the infinitesimal intervals along meridians reflecting more local corneal curvatures (shapes).

Wavefront aberrations and treatment zone after orthokeratology were measured with Nidek OPD-Scan III (Nidek Technologies, Gamagori, Japan) (based on automatic retinoscopy; provides integrated corneal topography and wavefront measurement in one device) [18]. Ocular and corneal wavefront aberrations for a 3, 4, or 5 mm optical zone across the undilated pupil were measured. Data were expanded with the normalized Zernike polynomials up to the eighth order. Magnitudes of the coefficients of the Zernike polynomials were represented as the root mean square (RMS). Total aberration, higher-order aberration, third- to eighth-order coefficient, astigmatism, spherical aberration, coma, trefoil, and tetrafoil were measured and analyzed separately. Horizontal and vertical diameters across the center of treatment zone (corneal refractive power within 45.0 D) were measured from the instantaneous map of cornea provided by OPD-Scan III (Figure 1) and the average was used. Measurements were repeated at least five times for each eye, and the three best-focused images were selected. The average values were used for subsequent analysis.

### 2.3. Contrast Sensitivity Function

CSF was measured under mesopic (2.7 cd/m^2^) and photopic (85 cd/m^2^) conditions, with and without high glare (CSV-1000E, VectorVision, Greenville Ohio, USA). CSF under mesopic conditions was measured in dark room and first tested without glare and then with glare. The combination of contrast and glare test was performed with halogen glare lights positioned at the sides of the console. The glare lights did not alter the illumination of the console. Monocular measurements were taken at 2.5 m distance with the best spectacle correction before orthokeratology and without correction after orthokeratology. The contrast threshold in logarithmic values for 3, 6, 12, and 18 cpd and the area under the log CSF (AULCSF) were used for subsequent analysis [19].

### 2.4. Subjective Questionnaire

Quality of Life Impact of Refractive Correction (QIRC) [20] was used to evaluate the subjective vision experience by the same ophthalmologist. Each subject was tested twice separately before and 1 month after orthokeratology.

### 2.5. Statistical Analysis

All statistical analyses were performed using SPSS 18.0 (SPSS Inc, Chicago, Illinois, USA). All continuous variables were expressed as the mean ± standard deviations (Mean ± SD). The normality of each variable was checked with the 1-sample Kolmogorov-Smirnov test. Comparisons of the parameters before and after orthokeratology and between morning and afternoon on the same day were performed by using a paired t-test. The level of significance was *P* less than 0.05.

## 3. Results

Among the 22 children who were enrolled at baseline, 3 of them dropped out of the study because their UCVA did not achieve 20/20 due to the decentration of orthokeratology lens. Among the 19 children, 4 subjects finished partial measurements due to poor cooperation.

One month after orthokeratology, LogMAR UCVA in the morning (−0.066 ± 0.09) was significantly improved from baseline (0.557 ± 0.23, *P* < 0.001) and did not differ from that in the afternoon (0.049 ± 0.05, *P*=0.083).

Table 2 showed manifest sphere refraction and corneal curvature significantly reduced after orthokeratology (*P* < 0.001). Regular astigmatism did not change significantly (*P*=0.155). The diurnal variation of corneal curvature was statistically significant (*P* < 0.001), but sphere refraction was not (*P*=0.568).

One month after orthokeratology, OSI significantly increased from 0.295 ±0.15 to 0.652 ± 0.31 (*P* < 0.001), TFM-OSI increased from 0.572 ± 0.29 to 1.212 ± 0.97 (*P* < 0.002), MTF cut-off, SR, and OVs decreased significantly (*P* < 0.033). The diurnal variation of these parameters was not significant (Table 3).

**Figure 2:**
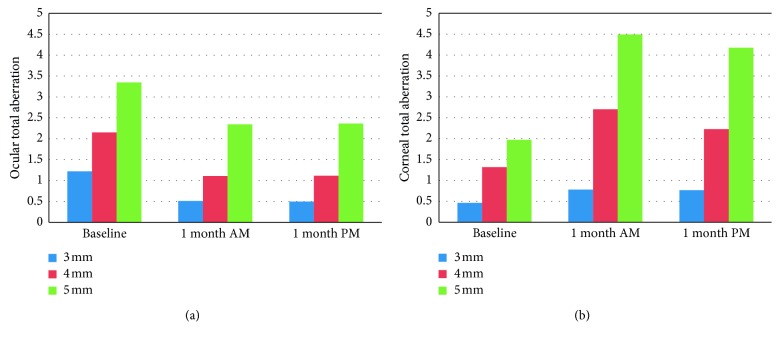
Effects of orthokeratology on ocular and corneal total aberrations (*n*=22  eyes).

**Figure 3:**
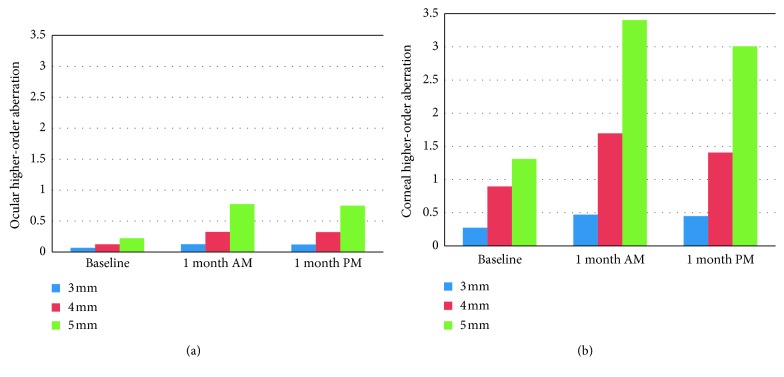
Effects of orthokeratology on ocular and corneal higher-order aberrations (*n*=22  eyes).

Tables 4 and 5 showed ocular and corneal aberrations respectively. One month after orthokeratology, ocular total aberration decreased significantly (*P* < 0.001). Ocular higher-order aberration, corneal total aberration, and corneal higher-order aberration increased significantly (*P* < 0.01) (Figures 2 and 3). Ocular and corneal coma, trefoil, tetrafoil, and spherical aberrations for 3, 4, and 5 mm optical zone increased significantly, except the ocular sixth- to eighth-order aberrations for 3 mm optical zone. The diurnal variation of aberrations was not significant, except ocular spherical aberration for 3 mm optical zone (*P*=0.03). Treatment zone diameters (TZD) decreased from 4.12 ± 0.18 mm to 3.95 ± 0.23 mm (*P*=0.001), and the average change was 0.16 ± 0.13 mm.

Changes in AULCSF under mesopic, photopic, and high glare conditions were not statistically significant before and after orthokeratology. The log CSF under photopic condition increased at 18 cpd (*P*=0.012), but decreased at 3 cpd (*P*=0.006). AULCSF under high glare and photopic conditions, log CSF at 18 cpd under mesopic and high glare conditions, log CSF at 12 cpd under photopic condition all increased significantly in the afternoon compared to the parameters in the morning at 1 month. Besides the above mentioned, no significant diurnal variation was found for other parameters of CSF (Table 6).

The survey of the subjective questionnaire showed that the dry eye symptom was more remarkable after orthokeratology (*P*=0.03), nevertheless the feeling of asthenopia was relieved (*P*=0.01). The mean score of satisfaction to orthokeratology was 92.25. During the whole day and night, self-reported vision was stable in 10 children (45%), 1 subject (5%) had a fluctuating vision, and 11 children (50%) reported that the vision in the morning was better than that in the evening.

## 4. Discussion

Orthokeratology can reduce the refractive error by remodeling the anterior surface of cornea temporarily [21]. With the improvement of refraction, the low-order aberrations, which constituted 80%∼85% of the ocular total aberration, reduced. Therefore, UCVA could be 20/20 or better after orthokeratology, as demonstrated in this study that most children whose best corrected visual acuity were 20/20 with spectacles before orthokeratology achieved 20/20 or better UCVA after 1 month of orthokeratology. Some research [22–24] indicated that orthokeratology could improve UCVA effectively. In addition, the increase of high spatial frequency CSF may be due to the improvement of UCVA after 1 month of orthokeratology because the high spatial frequency CSF mainly reflected the central macular vision. Furthermore, the improvement of vision and self-confidence after removal of spectacles as psychological and physiological factors may play a role. Nichols et al. [25] discovered that the changes of visual and refractive outcomes became stable around 1 month after orthokeratology. Soni et al. [23] even indicated that full effect of orthokeratology was achieved by the end of 1 week and remain stable for all waking hours of the day. Kang et al. [26] demonstrated that cornea experienced regression of correcting effects in the initial period of orthokeratology. This regression caused decline of visual acuity in the afternoon as corneal asphericity returns. However, the diurnal variation stabilized by 1 month. According to our results, the area of treatment zone at PM was smaller than that at AM, suggesting that the cornea had shape regression. Also, the diurnal variation of corneal curvature was statistically significant. However, the mean diurnal variation of flat and steep corneal curvature within 8 hours after lens removal was 0.27 D and 0.31 D, respectively. Taking into account that the axial length of normal eyes in the afternoon is shorter than that in the morning [27], the extent of diurnal variation of corneal shape after 1 month of orthokeratology had no influence on either manifest refraction or UCVA, indicating that orthokeratology was effective to improve UCVA and the effect was stable after 1 month of lens wear in myopic children.

However, the objective measurements revealed that the optical quality declined after orthokeratology. The value of MTF cut-off, SR, and OVs decreased. Overnight orthokeratology may cause midperipheral stromal thickening [28]. De Juan et al. [17] demonstrated that corneal swelling had a significant impact on the optical quality of the eye. The OSI significantly increased after orthokeratology. Jeon et al. [15] found that OSI increased after orthokeratology but still less than 1.0 on average, which is within the normal range [29]. This was consistent with our results and indicated that the visual quality can remain relatively good despite the slight increasing of intraocular scattering after orthokeratology. In our study, the mean value of OSI for all the myopic children was 0.29 ± 0.15 before orthokeratology, which was better than the result reported by Martínez-Roda et al. (0.38 ± 0.19) [29]. This may be due to the discrepancy of age distribution between the two studies. The intraocular scatter usually increased with age [30]. Furthermore, the TFM-OSI increased, illustrating that the stability of tear film decreased after orthokeratology. The results of subjective questionnaire survey also demonstrated that orthokeratology increases dry eye symptoms (photophobia, dryness, etc.). The stability of tear film also influenced the visual quality.

Ocular higher-order aberration, corneal total aberration, and corneal higher-order aberration increased after orthokeratology in this study. This was consistent with the previously published studies [4, 6, 31]. Corneal refractive therapy significantly increased spherical aberration in the positive direction with an impact on visual quality [32], which was also consistent with our results. It was reported that contrast sensitivity function after orthokeratology deteriorated in proportion to the increases in higher-order aberration [4]. As a consequence, the low spatial frequency CSF decreased, especially the decrease of log CSF at 3 cpd had statistical significance. The decrease of low spatial frequency CSF may be due to the midperipheral corneal steepening in the process of wearing orthokeratology, which affected the imaging function of peripheral retina. Hiraoka et al. [4] researched a group of myopic adults (46 eyes of 23 patients) undergoing overnight orthokeratology and evaluated the change of CSF. They found that orthokeratology treatment resulted in statistically significant decrease of CSF at all spatial frequencies, and AULCSF was significantly reduced from 1.451 ± 0.120 to 1.291 ± 0.177 (*P* < 0.0001). In the present study, the decrease of low spatial frequency CSF was consistent with the result of Hiraoka et al., but we found that AULCSF increased after orthokeratology and the high spatial frequency CSF increased in accordance with the improved UCVA [33]. Hiraoka et al. [34] mentioned that decentered orthokeratology lens could result in decreased CSF after treatment. All the subjects in our study who finished the follow-up were well fitted without obvious decentration of orthokeratology lenses, and this maybe the reason why the AULCSF did not decrease in this study. This indicated that orthokeratology influenced the low spatial frequency CSF, but did not compromise and even improve the high spatial frequency CSF. Lee et al. [35] reported that there were no statistically proved correlations between higher order aberrations and optical quality parameters (MTF cut-off and SR) for adults after refractive surgery. Whether the parameters of the myopic children with orthokeratology have the same outcomes needs further investigations.

In previous research, the corneal thickness [36], axial length, and intraocular pressure [37] showed diurnal changes in human eyes without orthokeratology treatment. Chakraborty et al. [38] indicated that ocular spherical aberration underwent statistically significant diurnal variation, i.e., spherical aberration was positive during the day and gradually became more negative toward the later afternoon/evening. They also found that the anterior corneal curvature was the flattest in the morning and gradually became steeper throughout the day, which led to a significant myopic refractive shift in spherical equivalent refraction later in the day, but it had an apparent paradoxical relationship with the fluctuation in axial length [27] (the longest axial length during the day and the shortest at night). All these physiological fluctuations may result in a compounded effect of visual quality in myopic children with orthokeratology treatment. In our study, the diurnal changes of objective parameters that already included the compounded influence of physiological fluctuations were stable. For 3 mm optical zone at 1 month, though the diurnal variation of ocular spherical aberration was significant (0.019 ± 0.016 *µ*m AM and 0.023 ± 0.011 *µ*m PM, *P*=0.03), corneal spherical aberration had no significant difference between the two time points. This indicated that the change of ocular spherical aberration was not induced by cornea. Furthermore, the corneal higher-order aberration had no change between the two time points. However, the parameter of the range beyond 5 mm was not measured, so the slight change in the central 3 mm optical zone could not exclude the effect of the change of corneal shape beyond 5 mm range. Berntsen et al. [6] studied 20 myopic adults and found that the change of spherical aberration did not play an important role in the increasing of higher-order aberration for a 3 mm pupil. So we inferred that the diurnal change of spherical aberration might have no clinical significance. The CSF at 1 month PM was slightly better than that at 1 month AM, especially the high spatial frequency CSF increased significantly. This may be due to the quick disappearance of corneal edema after lens removal [10, 17], while the refractive regression was not significant in the afternoon.

The change of optical quality of orthokeratology was a combination of the reduced refraction, the increased intraocular scattering, and the change of ocular and corneal aberrations. Any of the factors was independent and also interrelated to influence the different spatial frequency of CSF and UCVA. David et al. [39] suggested that LASIK provided better visual quality outcomes than orthokeratology for the treatment of low-to-moderate myopia. For myopic adults, considering exclusively the visual quality results, LASIK was a better treatment option than orthokeratology. However, the ablation procedure of refractive surgery may increase ocular scattering [35] and the procedure was irreversible. For myopic children, whose eyes had not yet stopped growing, orthokeratology would be the better choice because the effect of orthokeratology was reversible with regard to optical quality of the eye [40] and the corneal morphology [41]. Furthermore, orthokeratology was a safe option for myopia retardation [42]. Queiros et al. [43] found that orthokeratology achieved the best score among the four treatments (LASIK, spectacle, soft contact lens, and orthokeratology) in the satisfaction for correction and appearance. In the present study, the subjective questionnaire survey on myopic children after orthokeratology indicated that the satisfaction was relatively high, and only three of the children had a transient complaint of light distortion. Santolaria Sanz et al. [44] reported that light distortion tends to return to baseline after one week of treatment, suggesting that neural adaptation is capable of overcoming optical quality degradation. However, still 50% of children consciously thought night vision was worse compared to the vision in the morning and 1 subject (5%) had a fluctuating vision. According to our results, the value of MTF cut-off, SR, and OVs decreased and the high-order aberrations increased with the expanding of pupil diameter. This indicated that visual quality descends under dark environment with larger pupil. The poor night vision may due to the combined effects of more refractive regression and larger pupil diameter at night. More aberration and scattering also resulted in the decrease of the nighttime visual quality. This study did not involve the visual quality at night and the continuous change within the 8 hours during the day was not assessed. Further research was needed to investigate the relationship between the dynamic change of cornea and the change of visual quality after orthokeratology. As the visual quality after orthokeratology was a result of multiple factors, we should not only see the advantage that it can improve UCVA and control the progress of myopia but also consider the declined visual quality and the discomfort complained by children after orthokeratology. Scientific and objective attitude toward the popularity of orthokeratology could serve the clinical practice better.

## 5. Conclusions

Orthokeratology can effectively improve UCVA and high spatial frequency CSF by decreasing the low-order aberrations. However, MTF and CSF at low spatial frequency decreased because of the increase of intraocular scattering and high-order aberrations. Meanwhile, CSF at high spatial frequency fluctuates significantly at two times during the same day after 1 month orthokeratology. All these significant influence on children's vision provided valuable clues for future lens design and clinical practice.

## Figures and Tables

**Table 1:** The orthokeratology lens parameters in this study.

Brand	E&E	Euclid	Lucid
Origin	China	USA	Korea
Material	Boston XO	Boston equalens II	Boston XO
Dk (cm^2^/sec) (mL·O_2_/mL·mmHg) (ISO/fatt)	100 ∗ 10^–11^	90 ∗ 10^–11^	140 ∗ 10^–11^
The overall diameter (mm)	10.6	10.6	10.6
The optic zone diameter (mm)	6	6.2	6.2
The reverse curve (mm)	0.6	0.5	0.9
The anchor curve (mm)	1.3	1.2	0.8
The peripheral curve (mm)	0.4	0.5	0.5
The central thickness (mm)	0.22	0.22	0.23

**Table 2:** Effects of orthokeratology on refraction and corneal curvature (*n*=22  eyes, mean ± SD).

Time	Sphere (D)	Cylinder (D)	K1 (D)	K2 (D)
AM baseline	−3.83 ± 0.97	−0.47 ± 0.43	42.05 ± 1.26	43.19 ± 1.37
AM 1 month	−1.03 ± 0.85	−0.69 ± 0.57	39.75 ± 0.97	40.64 ± 1.27
PM 1 month	−1.10 ± 0.68	−0.66 ± 0.52	40.02 ± 1.05	40.95 ± 1.27
P1	<0.001	0.155	<0.001	<0.001
P2	0.568	0.500	<0.001	<0.001

Paired *t*-test. P1: comparison between AM baseline and AM 1 month after orthokeratology; P2: comparison between AM and PM during the same day 1 month after orthokeratology; K1: flat keratometric value; K2: steep keratometric value.

**Table 3:** Effects of orthokeratology on OSI, TFM-OSI, MTF cut-off, SR, and OVs (*n*=22  eyes, mean ± SD).

Time	OSI	TFM-OSI	MTF	SR	OVs-100%	OVs-20%	OVs-9%
*3 mm optical zone*							
AM baseline	—	—	48.332 ± 8.10	0.309 ± 0.07	1.611 ± 0.27	1.778 ± 0.40	1.922 ± 0.49
AM 1 month	—	—	38.812 ± 9.58	0.225 ± 0.07	1.294 ± 0.32	1.292 ± 0.41	1.316 ± 0.46
PM 1 month	—	—	42.225 ± 8.93	0.250 ± 0.07	1.408 ± 0.30	1.448 ± 0.42	1.489 ± 0.48
P1	—	—	<0.001	<0.001	<0.001	<0.001	<0.001
P2	—	—	0.067	0.064	0.068	0.053	0.068

*4 mm optical zone*							
AM baseline	0.295 ± 0.15	0.572 ± 0.29	46.089 ± 7.26	0.280 ± 0.05	1.537 ± 0.24	1.652 ± 0.35	1.733 ± 0.38
AM 1 month	0.652 ± 0.31	1.212 ± 0.97	37.312 ± 8.16	0.211 ± 0.05	1.244 ± 0.27	1.216 ± 0.33	1.233 ± 0.35
PM 1 month	0.712 ± 0.43	1.128 ± 0.59	38.856 ± 9.55	0.223 ± 0.06	1.295 ± 0.32	1.300 ± 0.41	1.325 ± 0.45
P1	<0.001	0.002	<0.001	<0.001	<0.001	<0.001	<0.001
P2	0.239	0.63	0.32	0.169	0.326	0.204	0.189

*5 mm optical zone*							
AM baseline	—	—	44.812 ± 8.87	0.275 ± 0.06	1.494 ± 0.30	1.602 ± 0.40	1.687 ± 0.46
AM 1 month	—	—	37.174 ± 7.96	0.215 ± 0.05	1.239 ± 0.27	1.209 ± 0.32	1.243 ± 0.36
PM 1 month	—	—	39.124 ± 10.37	0.224 ± 0.06	1.304 ± 0.35	1.322 ± 0.44	1.339 ± 0.44
P1	—	—	0.003	0.001	0.003	0.001	0.001
P2	—	—	0.177	0.294	0.178	0.051	0.109

Paired *t*-test. P1: comparison between AM baseline and AM 1 month after orthokeratology; P2: comparison between AM and PM during the same day 1 month after orthokeratology.

**Table 4:** Effects of orthokeratology on ocular aberrations (*n*=22  eyes, mean ± SD).

Time	Coma	Trefoil	Tetrafoil	Sph	S3	S4	S5	S6	S7	S8
*3 mm optical zone*										
Baseline AM	0.019 ± 0.01	0.044 ± 0.02	0.023 ± 0.01	−0.004 ± 0.01	0.050 ± 0.02	0.028 ± 0.01	0.017 ± 0.01	0.017 ± 0.01	0.013 ± 0.01	0.011 ± 0.01
1 month AM	0.049 ± 0.03	0.077 ± 0.04	0.037 ± 0.02	0.019 ± 0.02	0.099 ± 0.04	0.050 ± 0.02	0.029 ± 0.02	0.025 ± 0.02	0.018 ± 0.01	0.015 ± 0.01
1 month PM	0.052 ± 0.03	0.081 ± 0.05	0.030 ± 0.03	0.023 ± 0.01	0.101 ± 0.06	0.046 ± 0.03	0.027 ± 0.02	0.019 ± 0.01	0.015 ± 0.01	0.013 ± 0.01
P1	<0.001	0.002	0.005	<0.001	<0.001	<0.001	<0.001	0.11	0.081	0.235
P2	0.618	0.665	0.216	0.03	0.799	0.331	0.752	0.233	0.409	0.428

*4 mm optical zone*										
Baseline AM	0.043 ± 0.02	0.081 ± 0.03	0.035 ± 0.02	−0.010 ± 0.02	0.095 ± 0.03	0.047 ± 0.02	0.033 ± 0.01	0.028 ± 0.01	0.022 ± 0.01	0.018 ± 0.01
1 month AM	0.196 ± 0.09	0.136 ± 0.08	0.072 ± 0.03	0.099 ± 0.06	0.256 ± 0.10	0.143 ± 0.06	0.087 ± 0.04	0.060 ± 0.03	0.039 ± 0.03	0.033 ± 0.03
1 month PM	0.210 ± 0.08	0.145 ± 0.08	0.062 ± 0.05	0.108 ± 0.04	0.264 ± 0.10	0.141 ± 0.06	0.083 ± 0.05	0.046 ± 0.02	0.031 ± 0.02	0.026 ± 0.02
P1	<0.001	0.005	<0.001	<0.001	<0.001	<0.001	<0.001	0.001	0.007	0.017
P2	0.158	0.634	0.417	0.14	0.587	0.839	0.672	0.116	0.282	0.329

*5 mm optical zone*										
Baseline AM	0.091 ± 0.05	0.129 ± 0.04	0.059 ± 0.03	−0.019 ± 0.05	0.166 ± 0.05	0.091 ± 0.04	0.064 ± 0.03	0.047 ± 0.03	0.039 ± 0.02	0.032 ± 0.02
1 month AM	0.532 ± 0.26	0.223 ± 0.10	0.120 ± 0.06	0.333 ± 0.16	0.603 ± 0.26	0.393 ± 0.15	0.181 ± 0.09	0.112 ± 0.05	0.082 ± 0.05	0.065 ± 0.04
1 month PM	0.540 ± 0.24	0.222 ± 0.10	0.093 ± 0.07	0.328 ± 0.14	0.602 ± 0.24	0.373 ± 0.14	0.157 ± 0.08	0.095 ± 0.05	0.074 ± 0.04	0.051 ± 0.03
P1	<0.001	<0.001	<0.001	<0.001	<0.001	<0.001	<0.001	<0.001	0.001	0.002
P2	0.762	0.969	0.181	0.73	0.968	0.224	0.238	0.233	0.536	0.213

Paired *t*-test. P1: comparison between AM baseline and AM 1 month after orthokeratology; P2: comparison between AM and PM during the same day 1 month after orthokeratology.

**Table 5:** Effects of orthokeratology on corneal aberrations (*n*=22  eyes, mean ± SD).

Time	Coma	Trefoil	Tetrafoil	Sph	S3	S4	S5	S6	S7	S8
*3 mm optical zone*										
Baseline AM	0.048 ± 0.02	0.143 ± 0.05	0.105 ± 0.05	0.009 ± 0.01	0.155 ± 0.05	0.140 ± 0.05	0.091 ± 0.03	0.102 ± 0.03	0.063 ± 0.02	0.071 ± 0.02
1 month AM	0.120 ± 0.09	0.210 ± 0.14	0.171 ± 0.10	0.026 ± 0.03	0.251 ± 0.16	0.243 ± 0.16	0.162 ± 0.10	0.184 ± 0.12	0.104 ± 0.06	0.129 ± 0.09
1 month PM	0.109 ± 0.11	0.202 ± 0.14	0.170 ± 0.09	0.027 ± 0.02	0.242 ± 0.17	0.235 ± 0.15	0.149 ± 0.10	0.176 ± 0.12	0.097 ± 0.07	0.123 ± 0.09
P1	0.002	0.047	0.011	0.003	0.015	0.005	0.005	0.003	0.01	0.004
P2	0.663	0.836	0.946	0.924	0.825	0.81	0.616	0.77	0.666	0.761

*4 mm optical zone*										
Baseline AM	0.121 ± 0.05	0.452 ± 0.21	0.310 ± 0.11	0.038 ± 0.02	0.482 ± 0.19	0.413 ± 0.12	0.335 ± 0.11	0.314 ± 0.09	0.278 ± 0.12	0.221 ± 0.06
1 month AM	0.534 ± 0.39	0.673 ± 0.42	0.604 ± 0.35	0.198 ± 0.17	0.903 ± 0.53	0.820 ± 0.46	0.657 ± 0.32	0.641 ± 0.35	0.468 ± 0.26	0.440 ± 0.24
1 month PM	0.409 ± 0.40	0.561 ± 0.31	0.494 ± 0.34	0.200 ± 0.10	0.733 ± 0.49	0.696 ± 0.43	0.555 ± 0.30	0.533 ± 0.38	0.365 ± 0.25	0.357 ± 0.21
P1	<0.001	0.036	<0.001	<0.001	0.002	<0.001	<0.001	<0.001	0.004	<0.001
P2	0.056	0.192	0.181	0.923	0.101	0.237	0.12	0.192	0.055	0.149

*5 mm optical zone*										
Baseline AM	0.411 ± 0.37	0.439 ± 0.39	0.330 ± 0.32	0.099 ± 0.15	0.668 ± 0.46	0.490 ± 0.45	0.645 ± 0.45	0.366 ± 0.30	0.497 ± 0.27	0.253 ± 0.20
1 month AM	1.016 ± 0.42	1.175 ± 0.66	0.858 ± 0.53	0.451 ± 0.33	1.644 ± 0.62	1.454 ± 0.67	1.412 ± 0.69	1.266 ± 0.84	1.205 ± 0.83	1.042 ± 0.59
1 month PM	1.231 ± 1.39	0.956 ± 1.12	0.738 ± 0.71	0.408 ± 0.37	1.629 ± 1.77	1.388 ± 1.38	1.182 ± 1.28	0.993 ± 0.99	0.918 ± 0.92	0.823 ± 0.89
P1	<0.001	<0.001	<0.001	<0.001	<0.001	<0.001	<0.001	<0.001	0.001	<0.001
P2	0.474	0.443	0.528	0.636	0.971	0.844	0.485	0.346	0.296	0.292

Paired *t*-test. P1: comparison between AM baseline and AM 1 month after orthokeratology; P2: comparison between AM and PM during the same day 1 month after orthokeratology.

**Table 6:** Effects of orthokeratology on contrast sensitive function (*n*=22  eyes, mean ± SD).

Time	3 cpd	6 cpd	12 cpd	18 cpd	AULCSF
*Mesopic*					
AM baseline	1.620 ± 0.19	1.677 ± 0.17	1.552 ± 0.21	1.266 ± 0.37	1.245 ± 0.10
AM 1 month	1.606 ± 0.16	1.700 ± 0.26	1.573 ± 0.29	1.272 ± 0.36	1.257 ± 0.16
PM 1 month	1.632 ± 0.12	1.706 ± 0.21	1.551 ± 0.34	1.408 ± 0.38	1.267 ± 0.15
P1	0.706	0.634	0.7	0.913	0.659
P2	0.342	0.87	0.654	0.002	0.655

*High glare*					
AM baseline	1.636 ± 0.25	1.686 ± 0.24	1.483 ± 0.25	1.267 ± 0.37	1.235 ± 0.13
AM 1 month	1.577 ± 0.20	1.669 ± 0.32	1.517 ± 0.28	1.338 ± 0.35	1.235 ± 0.19
PM 1 month	1.613 ± 0.18	1.746 ± 0.22	1.562 ± 0.31	1.431 ± 0.38	1.285 ± 0.14
P1	0.19	0.787	0.514	0.233	0.992
P2	0.387	0.086	0.226	0.01	0.018

*Photopic*					
AM baseline	1.722 ± 0.17	1.657 ± 0.21	1.57 ± 0.29	1.266 ± 0.36	1.251 ± 0.13
AM 1 month	1.631 ± 0.14	1.716 ± 0.28	1.59 ± 0.27	1.397 ± 0.37	1.279 ± 0.17
PM 1 month	1.678 ± 0.14	1.775 ± 0.19	1.67 ± 0.22	1.430 ± 0.38	1.326 ± 0.11
P1	0.006	0.317	0.746	0.012	0.431
P2	0.115	0.129	0.017	0.371	0.01

Paired *t*-test. P1: comparison between AM baseline and AM 1 month after orthokeratology; P2: comparison between AM and PM during the same day 1 month after orthokeratology.
